# Subtherapeutic lithium supplementation causes physiological eccentric cardiac hypertrophy in young‐adult wild‐type male mice

**DOI:** 10.14814/phy2.70299

**Published:** 2025-04-02

**Authors:** Sophie I. Hamstra, Mia S. Geromella, Peter Tiidus, Panagiota Klentrou, Rebecca E. K. MacPherson, Val A. Fajardo

**Affiliations:** ^1^ Department of Kinesiology Brock University St. Catharines Ontario Canada; ^2^ Centre for Bone and Muscle Health Brock University St. Catharines Ontario Canada; ^3^ Department of Health Sciences Brock University St. Catharines Ontario Canada

**Keywords:** cardiac function, eccentric hypertrophy, GSK3, lithium, SERCA

## Abstract

Six weeks of low‐dose lithium (Li) supplementation has been shown to improve the activity of cardiac sarco(endo)plasmic reticulum calcium (Ca^2+^)‐ATPase (SERCA) in C57BL/6J wild‐type (WT) male mice. Improvements in myocardial SERCA function can lead to improvements in systolic and diastolic function in various rodent models. In this study, we tested the hypothesis that 12 weeks of subtherapeutic Li supplementation (10 mg/kg/day) would enhance SERCA function and positively influence cardiac contractility and morphology. Cardiac function and morphology were assessed using high‐frequency ultrasound in the final week of Li treatment. Subsequently, SERCA activity, Ca^2+^ uptake assays, and Western blotting for glycogen synthase kinase‐3β, SERCA2, and its inhibitor phospholamban (PLN) were performed on isolated left ventricle tissue. After 12 weeks of subtherapeutic Li supplementation, the heart underwent eccentric remodeling, exhibited by increased left ventricle internal diameter and volumes during systole and diastole, ultimately leading to greater stroke volume. However, we did not find any specific alterations in systolic or diastolic functional measures; nor were there any changes in SERCA activity and its content relative to PLN after Li supplementation. Thus, while Li supplementation appears to positively influence cardiac morphology to increase stroke volume, these changes are independent of changes to SERCA function.

## 1 INTRODUCTION

Cardiovascular diseases continue to be the leading cause of mortality globally, with aging, ischemic heart disease, hypertension, obesity, diabetes mellitus, and smoking being the leading causes of heart failure (Bozkurt et al., 2023; Vaduganathan et al., 2022). Dysregulation of the metabolic enzyme glycogen synthase kinase 3 (GSK3), particularly the GSK3β isoform, has been shown to contribute to cardiac dysfunction and progressive heart failure, making it a potential therapeutic target (Michael et al., 2004). One of the mechanisms in which dysregulated GSK3β contributes to cardiac dysfunction is through its negative regulation of the sarco(endo)plasmic reticulum calcium (Ca^2+^)‐ATPase (SERCA) in the myocardium (Flepisi et al., 2013). SERCA lies embedded within the membrane of the sarcoplasmic reticulum (SR) of cardiomyocytes and is responsible for over 70% of the reuptake of Ca^2+^ from the cytosol after each muscle contraction. In doing this, SERCA is a key regulator of muscle relaxation and thus, diastolic function in the heart. Additionally, by ensuring sufficient SR Ca^2+^ concentrations for subsequent contractions, SERCA also contributes to systolic function (MacLennan & Kranias, 2003). In human failing myocardium, abnormal intracellular calcium handling is seen with prolonged contractions and a diminished ability to restore resting low cytosolic Ca^2+^ concentrations during diastole (Gwathmey et al., 1987). Reduction in mRNA expression of SERCA2 has also been documented in failing human myocardium with various cardiomyopathies (Hasenfuss, 1998). SERCA2 has also been a well‐studied target for heart failure. Gene therapies aimed at increasing SERCA2 in the myocardium have shown promise in preclinical models (Byrne et al., 2008; Kawase et al., 2008; Sakata et al., 2007) however, these benefits did not translate to clinical trials in humans (Yla‐Herttuala, 2015). Therefore, finding alternative methods of maintaining or improving SERCA function in the heart would be important for the treatment and prevention of heart failure caused by systolic or diastolic dysfunction.

Given the relationship between GSK3 and cardiac SERCA function, developing strategies to inhibit GSK3 presents a promising alternative for enhancing SERCA performance and activity. In a study by Michael et al. (2004) they found that treating neonatal rat ventricular myocytes with LiCl (5 mM) alleviated the negative regulation of dysregulated GSK3β, thereby increasing SERCA2 mRNA expression. Li is a monovalent cation that is capable of inhibiting GSK3 both directly, via competition with its cofactor magnesium, and indirectly, via activation of the PI3K/Akt pathway, which phosphorylates GSK3 at the Ser 21 (GSK3α) and Ser 9 (GSK3β) sites, preventing substrate binding to the GSK3 isoforms (Hamstra, Whitley, et al., 2020). In our previous study, we demonstrated that treating 4–6 month‐old WT C57BL/6J male mice with low‐dose Li (10 mg/kg/day) for 6 weeks resulted in GSK3 inhibition in the left ventricle. Further, Li resulted in higher cardiac muscle SERCA2 content and apparent affinity of SERCA for Ca^2+^—the latter of which we attributed to an effect of Li on lowering the protein content of phospholamban (PLN) (Hamstra, Kurgan, et al., 2020). PLN is a small peptide, consisting of 52 amino acids, which physically interacts with SERCA to regulate its function by lowering its sensitivity for Ca^2+^ (Kimura et al., 1996; MacLennan & Kranias, 2003). This aligns well with our previous work using another GSK3 inhibitor, SB216763 (SB2), where treating Desmoglein‐2 mutant mice—a preclinical model of arrhythmogenic cardiomyopathy—preserved maximal ATPase activity by restoring SERCA2 and PLN levels to those of wild‐type mice (Hamstra et al., 2022). In that study, we also found that maximal SERCA activity positively correlated with ejection fraction and fractional shortening, further highlighting the benefits of GSK3 inhibition on cardiac contractility. However, whether such benefits to cardiac function and structure can also be observed with Li supplementation in mice remains unknown.

The aim of this study was to examine whether 12 weeks of subtherapeutic LiCl supplementation could affect cardiac structure and function in male C57BL/6J mice based on our previous results showing improvements in cardiac SERCA function with subtherapeutic LiCl supplementation for 6 weeks. Specifically, we hypothesized that the increased Ca^2+^ sensitivity and rise in the SERCA2:PLN ratio observed after 6 weeks of Li treatment would result in structural and functional changes linked to enhanced systolic and diastolic function with extended 12‐week treatment.

## 2 MATERIALS AND METHODS

### 2.1 Animals and study design

The hearts from this study were obtained from a previously published study from our lab, and full details on animal procedures can be reviewed in Geromella et al. (2022). To briefly summarize, male wild‐type C57BL/6J mice (16 weeks of age, *n* = 12) were supplemented for 12 weeks with low‐dose Li treatment (10 mg/kg/day dose in drinking water, serum [Li^+^] = 0.02 mM ± 0.004) (Hamstra, Kurgan, et al., 2020). Mice were housed individually and given free access to a chow diet (2014 Teklad global, 14% protein rodent maintenance diet, Harlan Teklad). For lithium supplementation, mice were randomly divided into control (*n* = 12) and lithium groups (*n* = 12). A stock bottle of 50 mg/mL lithium chloride (L4408; Sigma‐Aldrich; St. Louis, MO, USA) in distilled water was prepared for the lithium treatment group. Water bottles were filled twice a week, and stock lithium chloride (LiCl) solution was added to 200 mL of water to achieve a daily dose of 10 mg/kg body mass/day based on an average 5 mL consumption of water per day. The mice had free access to their water, and supplementation occurred for 12 weeks. Control water bottles contained 200 mL of water that was not supplemented with LiCl. Body mass, food intake, and water intake were measured twice a week. Mice were kept on a 12 h light/dark cycle and had ad libitum access to food and water. All procedures were approved by the Brock University Animal Care Committee (File #17‐06‐03) and were in compliance with the Canadian Council on Animal Care. Euthanasia was performed after 12 weeks of treatment by exsanguination under the administration of 5% isoflurane in oxygen.

### 2.2 High‐frequency ultrasound

To assess cardiac function and structure, all mice were subjected to high‐frequency ultrasound (HFU) analysis using a Prospect T1 high‐frequency ultrasound (S‐Sharp, Taiwan). Mice were given 3% isoflurane gas at an oxygen flow rate of 1 mL/min. Hair on the superior ventral surface was removed with a depilatory cream. Once the hair was removed, the mouse was placed on the HFU platform, with limbs taped to electrocardiogram (ECG) electrodes covered with ECG cream. A rectal probe was placed to monitor body temperature, and the heart rate was monitored and maintained at 400–500 bpm by adjusting the isoflurane gas delivery. The ventral surface was then covered with ultrasound gel to form an acoustic coupling with the ultrasound probe. Short‐axis M‐mode imaging was used to measure and calculate cardiac morphology and systolic function, including the left ventricle internal diameter (LVID), relative wall thickness (RWT), end‐diastolic volume (EDV), end‐systolic volume (ESV), stroke volume (SV), uncorrected and corrected left ventricular mass (LV Mass (U) and LV Mass (C), respectively), percent ejection fraction (%EF), percent fractional shortening (%FS), and cardiac output (CO). Pulse‐wave doppler of the mitral valve inflow from an apical 4‐chamber view was used to assess diastolic function, systolic function, and cardiac compliance, specifically measuring E wave velocity, A wave velocity, deceleration time, isovolumic relaxation time (IVRT), isovolumic contraction time (IVCT), and myocardial performance index (MPI) which is the sum of IVRT and IVCT divided by ejection time. Upon completion, mice were then removed from the platform, and the isoflurane gas was placed in a recovery cage on top of a warm heating pad until they woke up.

### 2.3 Tissue collection

After 12 weeks of treatment, mice were euthanized, and hearts were harvested and frozen in liquid N_2_ immediately to be stored at −80°C. Left ventricles were isolated from the frozen tissue and homogenized in a 10:1 ratio with homogenizing buffer (250 mM sucrose, 5 mM HEPES, 0.2 mM phenylmethylsulfonyl fluoride, 0.2% [w/v] NaN_3_ in distilled H_2_O, and pH 7.5).

### 2.4 Calcium uptake assay

Calcium uptake assay was run as described previously (Geromella et al., 2023). To summarize, Ca^2+^ uptake was measured in left ventricle homogenates using Ca^2+^ indicator Indo‐1 (50041; Biotum) measuring the difference between Ca^2+^‐bound Indo‐1 and Ca^2+^‐free Indo‐1. As Indo‐1 cannot cross the SR membrane, the amount of Ca^2+^‐bound Indo‐1 decreases as Ca^2+^‐free Indo‐1 increases. This is measured at two fluorescence emissions (450 nm for Ca^2+^‐bound Indo‐1 and 485 nm for Ca^2+^‐free Indo‐1) at 37°C. Each sample was plated in duplicate in an all‐black 96‐well plate with 1 μL of 2 mM Indo‐1 and Ca^2+^ uptake buffer (200 mM KCl, 20 mM HEPES, 10 mM NaN_3_, 5uM TPEN, 5 mM Oxalate, and 15 mM MgCl_2_) to read starting calcium concentrations in each sample. Then 4 μL of 250 mM ATP (A2383; Sigma) was added to each well to initiate Ca^2+^ uptake by activating the SERCA pump, and this reaction was read for 30 min. Subsequently, to calculate free intracellular Ca^2+^ concentration, we used a two‐point calibration system with the addition of 50 mM EGTA (E3889; Sigma) (low calcium), followed by 100 mM CaCl_2_ (C4901; Sigma) (high calcium) to each well. Free intracellular Ca^2+^ concentration was then calculated using the following formula:

$$\left[{\mathrm{Ca}}^{2+}\right]=K_{d}\left(\frac{R-R\min}{R\max-R}\right)\left(\frac{S_{f2}}{S_{b2}}\right)$$

where *K*_*d*_ is the dissociation constant of Indo‐1 at 250 nm. *R* is the ratio of Ca^2+^‐bound fluorescence (405 nm) to Ca^2+^‐free fluorescence (485 nm) for each timepoint. *R*_min_ is the ratio of bound: unbound Indo‐1 in the presence of EGTA. *R*_max_ is the ratio of bound: unbound Indo‐1 in the presence of high [Ca^2+^]. *S*_*f*2_ is the fluorescence emission of Ca^2+^‐free Indo‐1 at 485 nm. *S*_*b*2_ is the fluorescence emission of Ca^2+^‐free Indo‐1 in the presence of high [Ca^2+^] at 485 nm.

Rates of Ca^2+^ uptake were measured as a tangent or instantaneous velocity when the Ca^2+^ curve crossed 1500 nM free intracellular Ca^2+^ by plotting the calibrated kinetic data in LoggerPro (Vernier Software, Beaverton, OR, USA). All data was normalized to protein content using the BCA assay (B9643; Sigma).

### 2.5 ATPase activity assay

SERCA‐specific ATPase activity was measured using an ionophore (A23187; Sigma)‐supported spectrophotometric assay across a range of Ca^2+^ concentrations (pCa 7.2 to 6.0). This assay is an enzyme‐linked spectrophotometric assay fitted onto a 96‐well plate (Hamstra, Kurgan, et al., 2020). ATP hydrolysis was measured as a 1:1 ratio of NADH disappearance using LDH (18 U/L) (10127230001; Sigma) and PK (18 U/L) (0128155001; Sigma). Kinetic activity is read over 30 min at 340 nm using an M2 Molecular Devices MultiMode plate reader. SERCA‐specific ATPase activity was calculated, correcting for pathlength, using the extinction coefficient of NADH (6.22 mM) (10107735001; Sigma) after subtracting activity with the SERCA‐specific inhibitor cyclopiazonic acid (40 mM) (239805; Sigma) from all values. Data was normalized to grams of protein determined by a bicinchoninic acid assay. Kinetic data was fitted onto a sigmoidal dose–response curve to measure maximal ATPase activity and normalized to %Vmax to compare *p*Ca_50_, the amount of calcium required to reach 50% Vmax, using GraphPad Prism 8 software (GraphPad Software Inc., La Jolla, CA, USA).

### 2.6 Western blotting

Sample prep was prepared at 2 μg/μL protein in 4× Laemmli buffer (1610747; BioRad; Hercules, CA, USA) based on protein concentrations of each sample measured using a bicinchoninic acid (BCA) assay. Protein content was assessed by performing Western blotting for SERCA2, PLN, phosphorylated (Ser9) GSK3β, and total GSK3β. TGX BioRad Precast 4%–15% gradient gels (4561086; BioRad) were used for all proteins. Protein was transferred to PVDF membranes for SERCA2 and total GSK3β and nitrocellulose for pGSK3β and PLN using a BioRad Transblot Turbo. All membranes were blocked with EveryBlot blocking buffer (12010020; BioRad). SERCA2 (MA3‐919; ThermoFisher Scientific; Waltham, MA, USA), PLN (MA3‐922; ThermoFisher Scientific), and total GSK3β (9315; Cell Signaling Technology; Beverly, MA, USA) antibodies were incubated with 5% milk, while pGSK3β (Ser9; 9336; Cell Signaling Technology) antibody was incubated with 3% BSA. After primary incubations and Tris‐buffered saline with Tween‐20 (TBST) washes, membranes were incubated for1 h at room temperature with corresponding secondary antibodies (Anti‐mouse (SERAC2 and PLN—7076; Cell Signaling Technology); Anti‐rabbit (pGSK3β and tGSK3β—7074; Cell Signaling Technology)). After TBST washes, membranes were visualized with Millipore Immobilon chemiluminescent HRP substrate (WBKLS0500; Sigma‐Aldrich) on a BioRad Chemi Doc Imager. Images were analyzed using ImageLab (BioRad) to quantify optical densities, which were normalized to total protein visualized with ponceau stain (59803; Cell Signaling Technology).

### 2.7 Statistical analysis

To examine the effects of low‐dose LiCl treatment on cardiac function compared to control‐treated mice, a two‐tailed Student's *t*‐test was used for cardiac functional and morphological measures as well as protein content analysis with Welch's correction when necessary. A *p* value of *p* ≤ 0.05 was used to define statistical significance. A 5% ROUT test was used to identify outliers for all analyses. When identified, outliers were removed from statistical analyses. All statistical tests were conducted using GraphPad Prism 8 Software, and all data is presented as mean ± SD.

## 3 RESULTS

### 3.1 Subtherapeutic lithium causes eccentric hypertrophy of the left ventricle without altering systolic or diastolic function

Previous work has shown that 6 weeks of low‐dose LiCl can improve the SERCA2:PLN ratio and SERCA Ca^2+^ sensitivity in mouse cardiac tissue (Hamstra, Kurgan, et al., 2020). Improved cardiac SERCA function and the SERCA2:PLN ratio have been shown to affect systolic and diastolic function and promote physiological hypertrophy in disease conditions (Kranias & Hajjar, 2012; Makarewich et al., 2018); therefore, cardiac morphology, systolic, and diastolic function were assessed. Table 1 demonstrates that 12 weeks of subtherapeutic LiCl supplementation resulted in a higher internal diameter of the left ventricle at both peak systole and diastole. This higher internal diameter was not due to any change in anterior or posterior wall thickness, though LiCl treated mice tended to have lower relative wall thickness at peak systole (*p* = 0.06). Uncorrected and corrected LV Mass was also higher in LiCl supplemented mice compared to control. Corresponding well with the greater internal diameter, the LiCl group had higher ESV and EDV when compared to controls (Table 2). These changes led to a higher stroke volume for the LiCl group; however, no differences in cardiac output were detected. With respect to systolic function, there were no changes in ejection fraction or fractional shortening between groups (Table 2). Additionally, there were no differences in any diastolic parameters (Table 3). Collectively, these data suggest that 12 weeks of subtherapeutic LiCl treatment in male mice results in greater stroke volume by increasing LV chamber size, without affecting systolic or diastolic function.

**TABLE 1:** Eccentric remodeling occurs with 12‐week low‐dose lithium supplementation.

Measures	Control (*n* = 12)	LiCl (*n* = 10)
LVAW;d (mm)	1.05 ± 0.19	1.00 ± 0.09
LVAW;s (mm)	1.51 ± 0.23	1.47 ± 0.12
LVID;d (mm)	3.99 ± 0.27	4.32 ± 0.17**
LVID;s (mm)	2.74 ± 0.25	3.00 ± 0.25*
LVPW;d (mm)	0.85 ± 0.04	0.86 ± 0.07
LVPW;s (mm)	1.23 ± 0.06	1.20 ± 0.09
RWT;d (mm)	0.42 ± 0.03	0.40 ± 0.04
RWT;s (mm)	0.89 ± 0.09	0.80 ± 0.10^#^
LV Mass (U) (mg)	144 ± 18.35	164 ± 16.28*
LV Mass (C) (mg)	115 ± 14.68	131 ± 13.03*

*Note*: Morphological measures were taken in M‐mode imaging with a short axis view. Wall thickness of the anterior (AW) and posterior walls (PW) of the left ventricle was measured at peak systole (LVAW;s and LVPW;s, respectively) and diastole (LVAW;d and LVPW;d, respectively). Left ventricle internal diameter was also measured at peak systole (LVID;s) and diastole (LVID;d). Relative wall thickness during systole (RWT;s) and diastole (RWT;d) was calculated using the corresponding LVPW and LVID measures. Left ventricle mass was measured by ultrasound (uncorrected—LV Mass (U) and corrected—LV Mass (C)). All data is shown as mean ± SD.

^#^ *p <* 0.10, *p < 0.05, **p < 0.01 using a Student's *t‐*test.

**TABLE 2:** Low‐dose lithium supplementation increases left ventricular capacity without altering systolic function.

Measures	Control (*n* = 12)	LiCl (*n* = 10)
HR (bpm)	491 ± 43.59	475 ± 26.52
FS (%)	31.5 ± 3.69	30.7 ± 4.70
EF (%)	59.7 ± 5.33	59.7 ± 5.94
EDV (μL)	70.1 ± 10.64	84.4 ± 8.06**
ESV (μL)	28.3 ± 5.87	33.2 ± 4.11*
SV (μL)	41.8 ± 6.94	49.6 ± 7.48*
CO (mL/min)	20.5 ± 4.10	23.4 ± 3.86

*Note*: Systolic measures were also taken in M‐mode imaging with a short‐axis view. End‐systolic and end‐diastolic volumes (ESV and EDV, respectively) were calculated using the corresponding LVID measures. Fractional shortening (FS) was also calculated using LVID. ESV and EDV were used to calculate ejection fraction (EF) and stroke volume (SV). Heart rate (HR) and SV were used to calculate cardiac output (CO). All data is shown as mean ± SD.

*p < 0.05, **p < 0.01 using a Student's *t‐*test.

**TABLE 3:** Low‐dose lithium supplementation does not alter diastolic function.

Measures	Control (*n* = 12)	LiCl (*n* = 10)
A wave velocity (mm/s)	439 ± 116.8	475 ± 80.52
Acceleration (m/s^2^)	66.2 ± 9.76	76.2 ± 28.13
Deceleration time (ms)	25.9 ± 7.62	26.4 ± 7.49
E wave velocity (mm/s)	531 ± 74.52	525 ± 123.2
E/A	1.26 ± 0.24	1.11 ± 0.20
Ejection time (ms)	39.7 ± 7.02	39.0 ± 4.02
IVCT (ms)	11.9 ± 3.82	13.8 ± 5.06
IVRT (ms)	17.1 ± 2.46	15.5 ± 2.61
MPI	0.76 ± 0.19	0.77 ± 0.18

*Note*: Pulse‐wave Doppler imaging with an apical view was used to measure blood flow from the mitral valve into the left ventricle. Peak velocity of the early (E wave) and late (A wave) phases of ventricular filling along with the deceleration time of the E wave and acceleration of the A wave were measured. The E/A ratio was calculated using the E and A wave velocities. Myocardial performance index (MPI) was measured using the isovolumic contraction time (IVCT) and isovolumic relaxation time (IVRT). All data is presented as mean ± SD.

*p < 0.05, **p < 0.01 using a Student's *t‐*test.

### 3.2 SERCA2:PLN ratio increases with subtherapeutic LiCl

**FIGURE 1:**
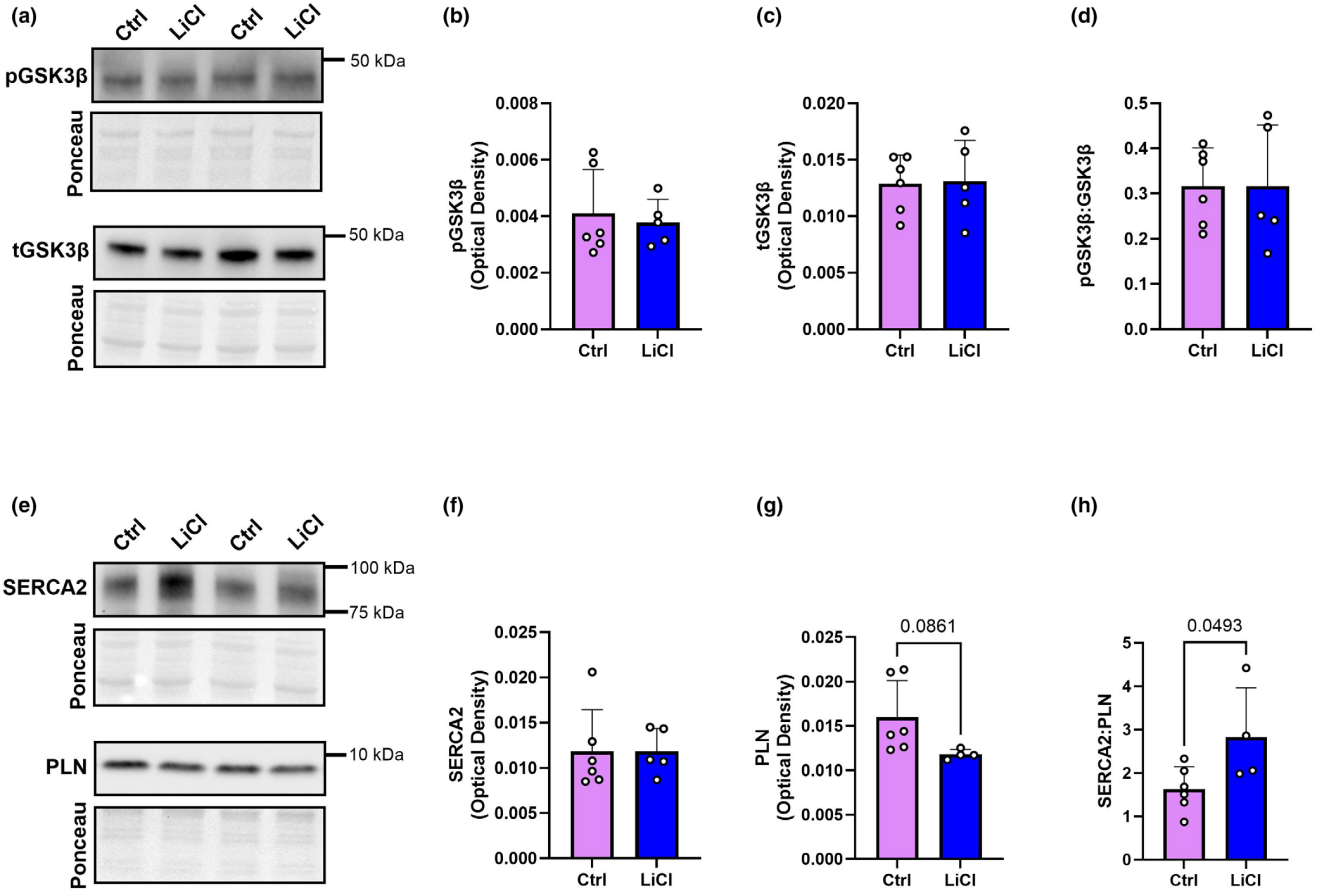
GSK3, SERCA, and PLN content are unchanged with 12 weeks LiCl treatment. Representative Western blots of phosphorylated (Ser9) GSK3β (pGSK3β) and total GSK3β (tGSK3β) (a). Optical density analysis of pGSK3β (b), tGSK3β (c), and their ratio (d). Representative blots of SERCA2 and PLN (e). Optical density analysis of SERCA2 (f), PLN (g), and their ratio (h). For (b–d) and (f–h), a two‐tailed Student's *t*‐test was used with exact *p* values shown for statistically significant (*p* < 0.05) or trending (*p* < 0.10) comparisons. All values represented as mean ± SD, *n* = 5–6 per group.

As LiCl is an inhibitor of GSK3β, a key regulator of cardiac hypertrophy, it was important to assess its activity through Western blot measurement of total and Ser9 phosphorylation (inhibited) state (Hamstra, Whitley, et al., 2020; Michael et al., 2004). It was also important to confirm whether the greater SERCA2:PLN ratio observed in our previous 6‐week study with subtherapeutic LiCl (Hamstra, Kurgan, et al., 2020) was still present after 12 weeks of treatment. Figure 1 shows no differences in GSK3β content or Ser9 phosphorylation of GSK3β between control and LiCl (Figure 1a–d). When assessing SERCA2 content, no differences are shown between groups (Figure 1e,f); however, the protein content of the SERCA2 inhibitor, PLN, is trending lower with subtherapeutic LiCl supplementation compared to the control group (Figure 1g). This led to a significantly greater ratio of SERCA2:PLN in the LiCl group versus the control group (Figure 1h). In summary, 12‐week subtherapeutic LiCl did not change GSK3β phosphorylation status but did lead to a greater SERCA2:PLN ratio through reductions in PLN protein content.

### 3.3 Subtherapeutic LiCl does not alter SERCA‐mediated Ca^2+^ uptake or ATPase function after 12 weeks

**FIGURE 2:**
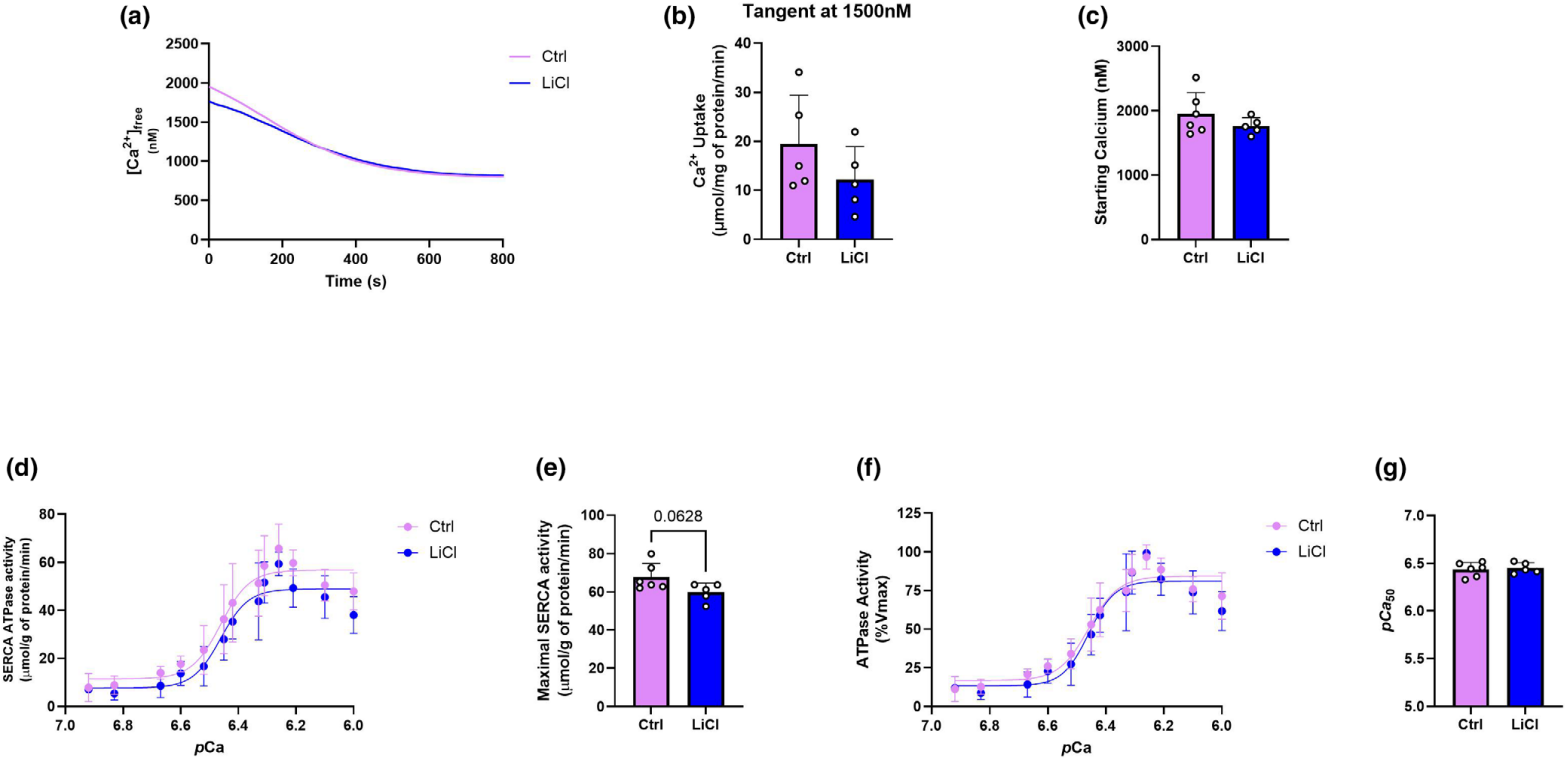
ATPase and Ca^2+^ uptake of the cardiac SERCA protein. Representative calcium uptake traces of Ctrl and LiCl‐treated left ventricle homogenates showing reductions in cytosolic calcium concentrations over time (a). Rate of SERCA‐mediated calcium uptake measured at 1500 nM Ca^2+^_free_ (b). Free calcium concentrations in both groups at the start of the assay (c). SERCA‐specific ATPase activity in left ventricle homogenates over increasing calcium concentrations represented as pCa (−log[Ca^2+^]) (d). Maximal activity achieved by SERCA (e). ATPase activity represented as a percent of maximal activity (%Vmax) (f). Calcium concentration required for SERCA to reach 50% of maximal activity represented as *p*Ca_50_ (g). For (b–c) and (e–f), a two‐tailed Student's *t*‐test was used with exact *p* values shown for statistically significant (*p* < 0.05) or trending (*p* < 0.10) comparisons. All values are represented as mean ± SD, *n* = 5–6 per group.

Given the increase in the SERCA2:PLN ratio, we next wanted to assess SERCA‐mediated Ca^2+^ uptake and ATPase activity to determine if SERCA function would be improved after 12 weeks of LiCl treatment. Furthermore, improvements to SERCA activity and its Ca^2+^ sensitivity via alterations in the SERCA2:PLN ratio can play a role in the regulation of cardiac hypertrophy and remodeling (Grote Beverborg et al., 2021; Makarewich et al., 2018). Figure 2 shows that SERCA‐mediated calcium uptake assays revealed no differences in the rate of Ca^2+^ uptake at 1500 nM Ca^2+^ or starting calcium concentration (Figure 2a–c). There was also a trending lower maximal ATPase activity in the LiCl group compared to control (Figure 2d,e) but there are no differences seen in SERCA *p*Ca_50_ (Figure 2f,g). Thus, although changes were seen in the amount of SERCA2 relative to its inhibitor PLN, 12 weeks of subtherapeutic LiCl did not have an effect on SERCA‐mediated Ca^2+^ uptake, activity, or its apparent affinity for Ca^2+^.

## 4 DISCUSSION

This study aimed to expand upon our laboratory's previous findings, which demonstrated that 6 weeks of low‐dose Li feeding increases SERCA2 content and its apparent affinity for Ca^2+^ (Hamstra, Kurgan, et al., 2020). The current investigation examined the effects of 12 weeks of LiCl feeding on cardiac morphology and function in male C57BL/6J mice, in addition to assessing SERCA function. We hypothesized that a longer‐term treatment of 12 weeks would be enough to elucidate any cardiac structural or functional changes that may be caused by the results of our treatment. Partly consistent with our hypothesis, we found that LiCl supplementation altered cardiac morphology, with an increase in LV mass, LVID, EDV, ESV, and SV; however, these effects were not met with any changes in SERCA activity.

Previous studies have demonstrated that GSK3 inhibition in muscle (both cardiac and skeletal muscle) can increase muscle size (Baranowski et al., 2023; Haq et al., 2000; Morisco et al., 2000; Pansters et al., 2015). Consistent with this literature, Table 1 shows higher LV mass in the LiCl group, suggesting hypertrophic remodeling. Along with higher LV mass, internal diameter during systole and diastole was also higher with LiCl supplementation without any differences in anterior or posterior wall thickness (Table 1), which leads us to conclude that 12 weeks of LiCl supplementation could be causing eccentric hypertrophy of the heart in mice. This apparent eccentric hypertrophy was not met with any alterations in systolic function (Tables 1 and 2) or diastolic function (Table 3). The lack of changes to systolic measures at rest, such as %FS, %EF, or CO (Table 2), is similar to the cardiac functional differences seen in endurance‐trained athletes at rest (Cumming, 1975; Fernandes et al., 2011, 2015; Vella & Robergs, 2005; Weeks & McMullen, 2011). Athletes have been shown to have greater ventricular dimensions and stroke volume at rest, which gives them the ability to have a greater increase in cardiac output during exercise when heart rate is also increased to meet the greater oxygen demands of the body (Cumming, 1975; Degens et al., 2019). In our previous study looking at the metabolic activity of these mice, we saw a greater level of energy expenditure (both absolute and relative to lean mass) during the 11th week of treatment in these LiCl‐supplemented mice without any significant changes in daily food intake, cage ambulation, or body mass (Geromella et al., 2022). It is possible that the changes in metabolic demand of the body caused by low‐dose Li supplementation for 12 weeks could cause these cardiac morphological changes. That is, an increase in metabolic activity heightens the demand for blood supply, leading to morphological changes that support an increase in stroke volume and cardiac output (Young, 2010); though the latter was not significantly elevated with LiCl supplementation (*p =* 0.1128). The lack of statistical significance is attributed to the LiCl group having a lower, albeit not statistically significant, heart rate compared with the control (491 bpm control vs. 475 bpm LiCl, *p* = 0.32). However, it is important to note that these measures were conducted at rest, and since endurance athletes have greater cardiac reserve and ability to increase cardiac output during exercise (Baggish & Wood, 2011), future experiments should consider assessing exercise performance in mice treated with subtherapeutic LiCl. Combined with our previous findings demonstrating that LiCl supplementation enhanced skeletal muscle endurance (Whitley et al., 2020) in mice, the eccentric hypertrophy seen here may also contribute to enhanced endurance performance in mice.

With respect to our Western blot analysis and similar to our previous results in the 6‐week study (Hamstra, Kurgan, et al., 2020), we did not see any changes in GSK3β content or its Ser9 phosphorylation status (Figure 1a–d). Importantly, Li is able to inhibit GSK3 both indirectly by activating the PI3K/Akt pathway to phosphorylate the Ser9 site of GSK3β and directly by competing with the cofactor magnesium given its similar ionic radius compared to Li (Freland & Beaulieu, 2012). Therefore, no differences in Ser9 phosphorylation do not necessarily mean GSK3 activity is not inhibited with our treatment as our previous work showed no changes in Ser9 phosphorylation but a 47% reduction in the ATPase activity of GSK3 measured using an enzymatic activity assay (Hamstra et al., 2023; Hamstra, Kurgan, et al., 2020; Hamstra, Whitley, et al., 2020). When looking at SERCA2 and PLN content, we see no changes in SERCA2 content; however, a trending reduction in PLN content (*p* = 0.0861) led to a significant increase in the SERCA2:PLN ratio (Figure 1e–h), a known determinant of cardiac contractility (MacLennan & Kranias, 2003). In our previous study, we saw a significant increase in SERCA2 and a decrease in PLN, leading to an increase in this ratio (Hamstra, Kurgan, et al., 2020), which can contribute to improvements in cardiac contractility and relaxation (Kranias & Hajjar, 2012). However, the increase in SERCA2 relative to its inhibitor PLN also explained the improvements we saw previously in SERCA Ca^2+^ sensitivity, as PLN normally reduces SERCA's affinity for Ca^2+^ (Hamstra, Kurgan, et al., 2020). Here, despite seeing a significant increase in the SERCA2:PLN ratio, we found no changes in SERCA‐mediated Ca^2+^ uptake or Ca^2+^ sensitivity (Figure 2). This could suggest that the SERCA pump has adapted to the LiCl in this 12‐week treatment period. Thus, our findings are only partly consistent with our previous work and suggest that the trending reduction in PLN content ultimately had no influence on SERCA function. Our results also revealed a trending reduction in maximal SERCA activity with LiCl supplementation (*p* = 0.0628) (Figure 2e); however, this did not have any negative effect on left ventricle diastolic function (Table 3). Overall, this data suggests that the changes observed in cardiac morphology must be independent of changes to SERCA function.

In any event, the results from this study suggest that low‐dose Li treatment for 12 weeks in C57 male mice does not appear to cause any detriment to SERCA2 content, the rate of Ca^2+^ uptake in the heart, or cause any negative changes to diastolic or systolic function. These findings are important as we do see positive effects of this dose of LiCl in other tissues such as bone (Kurgan, Bott, et al., 2019), skeletal muscle (Geromella et al., 2022; Kurgan, Whitley, et al., 2019; Whitley et al., 2020), and adipose while also increasing absolute energy expenditure (Geromella et al., 2022). Additionally, while lithium is recognized as the most effective mood stabilizer for bipolar disorder, its usage has decreased due to the emergence of other mood‐stabilizing medications and concerns over lithium toxicity, particularly beyond its limited therapeutic range of 0.5–1.2 mM in serum (Hamstra et al., 2023; Kessing, 2024; Richardson & Macaluso, 2017). In fact, L'Abbate et al. show that 12 weeks of supplementation with lithium carbonate (Li_2_CO_3_) at a therapeutic dose (43 mg/kg body weight; 1.73 mEq/L serum concentration) led to reductions in stroke volume and fractional shortening, increased cardiomyocyte cross sectional area (CSA) and alterations to cardiac electrical activity and arrhythmia susceptibility (L'Abbate et al., 2023). However, other studies have shown that subtherapeutic doses of Li can be cardioprotective by promoting physiological hypertrophy and recovery of ventricular function after ischemia/reperfusion injury (Chen et al., 2019; Faghihi et al., 2008) and reducing atherosclerotic lesions in a mouse model of high‐fat diet‐induced atherosclerosis (Choi et al., 2010). Thus, our data is more aligned with previous studies that demonstrate the beneficial effects of subtherapeutic Li treatment on cardiac function and morphology.

In conclusion, our findings indicate that low‐dose LiCl supplementation over a 12‐week period can induce an increase in left ventricular dimensions and stroke volume, resembling physiological eccentric hypertrophy. These changes could be due to increases in energy expenditure seen in these mice from our previously published study and not due to any changes in SERCA function or content as originally hypothesized. Future studies should confirm whether this remains true for C57BL/6J female mice and investigate any long‐term effects of this subtherapeutic treatment and its potential effects on exercise performance in mice.

## AUTHOR CONTRIBUTIONS

S.I.H. was involved in concept design, writing and drafting the manuscript, data collection, data interpretation, and reviewing and revising the manuscript. M.S.G. was involved in data collection and reviewing and revising the manuscript. P.T. and N.K. were involved in supervision and reviewing and revising the manuscript. R.E.K.M. was involved in the provision of reagents and resources, supervision, and reviewing and revising the manuscript. V.A.F. was involved in concept design, provision of reagents and resources, acquiring funding, supervision, and writing and drafting the manuscript.

## FUNDING INFORMATION

This work was supported by a Natural Sciences and Engineering Research Council (NSERC) Discovery Grant and a Canada Research Chair Tier II in Tissue Plasticity and Remodeling, both awarded to VAF. SIH is supported by an NSERC Vanier Canada Graduate Scholarship (CGS).

## CONFLICT OF INTEREST STATEMENT

The authors have no conflicts of interest to declare.

## ETHICS STATEMENT

All procedures perfromed in this study were approved by the Brock University Research Ethics Board and Animal Care Committee (File #17‐06‐03). All procedures were in compliance with the Canadian Council on Animal Care.

## Data Availability

All data supporting the results presented in this manuscript can be made available upon reasonable request.

## References

[phy270299-bib-0001] Baggish, A. L. , & Wood, M. J. (2011). Athlete's heart and cardiovascular care of the athlete: Scientific and clinical update. Circulation, 123, 2723–2735. 21670241 10.1161/CIRCULATIONAHA.110.981571

[phy270299-bib-0002] Baranowski, R. W. , Braun, J. L. , Hockey, B. L. , Yumol, J. L. , Geromella, M. S. , Watson, C. J. F. , Kurgan, N. , Messner, H. N. , Whitley, K. C. , MacNeil, A. J. , Gauquelin‐Koch, G. , Bertile, F. , Gittings, W. , Vandenboom, R. , Ward, W. E. , & Fajardo, V. A. (2023). Toward countering muscle and bone loss with spaceflight: GSK3 as a potential target. iScience, 26, 107047. 37360691 10.1016/j.isci.2023.107047 PMC10285634

[phy270299-bib-0003] Bozkurt, B. , Ahmad, T. , Alexander, K. M. , Baker, W. L. , Bosak, K. , Breathett, K. , Fonarow, G. C. , Heidenreich, P. , Ho, J. E. , Hsich, E. , Ibrahim, N. E. , Jones, L. M. , Khan, S. S. , Khazanie, P. , Koelling, T. , Krumholz, H. M. , Khush, K. K. , Lee, C. , Morris, A. A. , … Writing Committee Members . (2023). Heart failure epidemiology and outcomes statistics: A report of the Heart Failure Society of America. Journal of Cardiac Failure, 29, 1412–1451. 37797885 10.1016/j.cardfail.2023.07.006 PMC10864030

[phy270299-bib-0004] Byrne, M. J. , Power, J. M. , Preovolos, A. , Mariani, J. A. , Hajjar, R. J. , & Kaye, D. M. (2008). Recirculating cardiac delivery of AAV2/1SERCA2a improves myocardial function in an experimental model of heart failure in large animals. Gene Therapy, 15, 1550–1557. 18650850 10.1038/gt.2008.120

[phy270299-bib-0005] Chen, P. H. , Chao, T. F. , Kao, Y. H. , & Chen, Y. J. (2019). Lithium interacts with cardiac remodeling: The fundamental value in the pharmacotherapy of bipolar disorder. Progress in Neuro‐Psychopharmacology & Biological Psychiatry, 88, 208–214. 30053574 10.1016/j.pnpbp.2018.07.018

[phy270299-bib-0006] Choi, S. E. , Jang, H. J. , Kang, Y. , Jung, J. G. , Han, S. J. , Kim, H. J. , Kim, D. J. , & Lee, K. W. (2010). Atherosclerosis induced by a high‐fat diet is alleviated by lithium chloride via reduction of VCAM expression in ApoE‐deficient mice. Vascular Pharmacology, 53, 264–272. 20888430 10.1016/j.vph.2010.09.004

[phy270299-bib-0007] Cumming, G. R. (1975). Cardiac stroke volume: Effects of athletic training. The Journal of Sports Medicine, 3, 18–24. 1195695 10.1177/036354657500300104

[phy270299-bib-0008] Degens, H. , Stasiulis, A. , Skurvydas, A. , Statkeviciene, B. , & Venckunas, T. (2019). Physiological comparison between non‐athletes, endurance, power and team athletes. European Journal of Applied Physiology, 119, 1377–1386. 30919126 10.1007/s00421-019-04128-3

[phy270299-bib-0009] Faghihi, M. , Mirershadi, F. , Dehpour, A. R. , & Bazargan, M. (2008). Preconditioning with acute and chronic lithium administration reduces ischemia/reperfusion injury mediated by cyclooxygenase not nitric oxide synthase pathway in isolated rat heart. European Journal of Pharmacology, 597, 57–63. 18789320 10.1016/j.ejphar.2008.08.010

[phy270299-bib-0010] Fernandes, T. , Barauna, V. G. , Negrao, C. E. , Phillips, M. I. , & Oliveira, E. M. (2015). Aerobic exercise training promotes physiological cardiac remodeling involving a set of microRNAs. American Journal of Physiology. Heart and Circulatory Physiology, 309, H543–H552. 26071549 10.1152/ajpheart.00899.2014 PMC4537939

[phy270299-bib-0011] Fernandes, T. , Hashimoto, N. Y. , Magalhaes, F. C. , Fernandes, F. B. , Casarini, D. E. , Carmona, A. K. , Krieger, J. E. , Phillips, M. I. , & Oliveira, E. M. (2011). Aerobic exercise training‐induced left ventricular hypertrophy involves regulatory MicroRNAs, decreased angiotensin‐converting enzyme‐angiotensin ii, and synergistic regulation of angiotensin‐converting enzyme 2‐angiotensin (1‐7). Hypertension, 58, 182–189. 21709209 10.1161/HYPERTENSIONAHA.110.168252 PMC3184458

[phy270299-bib-0012] Flepisi, T. B. , Lochner, A. , & Huisamen, B. (2013). The consequences of long‐term glycogen synthase kinase‐3 inhibition on normal and insulin resistant rat hearts. Cardiovascular Drugs and Therapy, 27, 381–392. 23820981 10.1007/s10557-013-6467-8

[phy270299-bib-0013] Freland, L. , & Beaulieu, J.‐M. (2012). Inhibition of GSK3 by lithium, from single molecules to signaling networks. Frontiers in Molecular Neuroscience, 5, 14. 22363263 10.3389/fnmol.2012.00014 PMC3282483

[phy270299-bib-0014] Geromella, M. S. , Braun, J. L. , & Fajardo, V. A. (2023). Measuring SERCA‐mediated calcium uptake in mouse muscle homogenates. STAR Protocols, 4, 101987. 36602905 10.1016/j.xpro.2022.101987 PMC9826970

[phy270299-bib-0015] Geromella, M. S. , Ryan, C. R. , Braun, J. L. , Finch, M. S. , Maddalena, L. A. , Bagshaw, O. , Hockey, B. L. , Moradi, F. , Fenech, R. K. , Ryoo, J. , Marko, D. M. , Dhaliwal, R. , Sweezey‐Munroe, J. , Hamstra, S. I. , Gardner, G. , Silvera, S. , Vandenboom, R. , Roy, B. D. , Stuart, J. A. , … Fajardo, V. A. (2022). Low‐dose lithium supplementation promotes adipose tissue browning and sarco(endo)plasmic reticulum Ca(2+) ATPase uncoupling in muscle. The Journal of Biological Chemistry, 298, 102568. 36209826 10.1016/j.jbc.2022.102568 PMC9664358

[phy270299-bib-0016] Grote Beverborg, N. , Später, D. , Knöll, R. , Hidalgo, A. , Yeh, S. T. , Elbeck, Z. , Silljé, H. H. , Eijgenraam, T. R. , Siga, H. , & Zurek, M. (2021). Phospholamban antisense oligonucleotides improve cardiac function in murine cardiomyopathy. Nature Communications, 12, 5180. 10.1038/s41467-021-25439-0 PMC8405807 34462437

[phy270299-bib-0017] Gwathmey, J. K. , Copelas, L. , MacKinnon, R. , Schoen, F. J. , Feldman, M. D. , Grossman, W. , & Morgan, J. P. (1987). Abnormal intracellular calcium handling in myocardium from patients with end‐stage heart failure. Circulation Research, 61, 70–76. 3608112 10.1161/01.res.61.1.70

[phy270299-bib-0018] Hamstra, S. I. , Braun, J. L. , Chelko, S. P. , & Fajardo, V. A. (2022). GSK3‐inhibition improves maximal SERCA activity in a murine model of arrhythmogenic cardiomyopathy. Biochimica et Biophysica Acta ‐ Molecular Basis of Disease, 1868, 166536. 36057371 10.1016/j.bbadis.2022.166536

[phy270299-bib-0019] Hamstra, S. I. , Kurgan, N. , Baranowski, R. W. , Qiu, L. , Watson, C. J. F. , Messner, H. N. , MacPherson, R. E. K. , MacNeil, A. J. , Roy, B. D. , & Fajardo, V. A. (2020). Low‐dose lithium feeding increases the SERCA2a to phospholamban ratio improving SERCA function in murine left ventricles. Experimental Physiology, 105, 666–675. 32087034 10.1113/EP088061

[phy270299-bib-0020] Hamstra, S. I. , Roy, B. D. , Tiidus, P. , MacNeil, A. J. , Klentrou, P. , MacPherson, R. E. K. , & Fajardo, V. A. (2023). Beyond its psychiatric use: The benefits of low‐dose lithium supplementation. Current Neuropharmacology, 21, 891–910. 35236261 10.2174/1570159X20666220302151224 PMC10227915

[phy270299-bib-0021] Hamstra, S. I. , Whitley, K. C. , Baranowski, R. W. , Kurgan, N. , Braun, J. L. , Messner, H. N. , & Fajardo, V. A. (2020). The role of phospholamban and GSK3 in regulating rodent cardiac SERCA function. American Journal of Physiology. Cell Physiology, 319, C694–C699. 32755452 10.1152/ajpcell.00318.2020

[phy270299-bib-0022] Haq, S. , Choukroun, G. , Kang, Z. B. , Ranu, H. , Matsui, T. , Rosenzweig, A. , Molkentin, J. D. , Alessandrini, A. , Woodgett, J. , Hajjar, R. , Michael, A. , & Force, T. (2000). Glycogen synthase kinase‐3beta is a negative regulator of cardiomyocyte hypertrophy. The Journal of Cell Biology, 151, 117–130. 11018058 10.1083/jcb.151.1.117 PMC2189812

[phy270299-bib-0023] Hasenfuss, G. (1998). Alterations of calcium‐regulatory proteins in heart failure. Cardiovascular Research, 37, 279–289. 9614485 10.1016/s0008-6363(97)00277-0

[phy270299-bib-0024] Kawase, Y. , Ly, H. Q. , Prunier, F. , Lebeche, D. , Shi, Y. , Jin, H. , Hadri, L. , Yoneyama, R. , Hoshino, K. , Takewa, Y. , Sakata, S. , Peluso, R. , Zsebo, K. , Gwathmey, J. K. , Tardif, J. C. , Tanguay, J. F. , & Hajjar, R. J. (2008). Reversal of cardiac dysfunction after long‐term expression of SERCA2a by gene transfer in a pre‐clinical model of heart failure. Journal of the American College of Cardiology, 51, 1112–1119. 18342232 10.1016/j.jacc.2007.12.014

[phy270299-bib-0025] Kessing, L. V. (2024). Why is lithium [not] the drug of choice for bipolar disorder? A controversy between science and clinical practice. International Journal of Bipolar Disorders, 12, 3. 38228882 10.1186/s40345-023-00322-7 PMC10792154

[phy270299-bib-0026] Kimura, Y. , Kurzydlowski, K. , Tada, M. , & MacLennan, D. H. (1996). Phospholamban regulates the Ca2+‐ATPase through intramembrane interactions. The Journal of Biological Chemistry, 271, 21726–21731. 8702967 10.1074/jbc.271.36.21726

[phy270299-bib-0027] Kranias, E. G. , & Hajjar, R. J. (2012). Modulation of cardiac contractility by the phospholamban/SERCA2a regulatome. Circulation Research, 110, 1646–1660. 22679139 10.1161/CIRCRESAHA.111.259754 PMC3392125

[phy270299-bib-0028] Kurgan, N. , Bott, K. N. , Helmeczi, W. E. , Roy, B. D. , Brindle, I. D. , Klentrou, P. , & Fajardo, V. A. (2019). Low dose lithium supplementation activates Wnt/beta‐catenin signalling and increases bone OPG/RANKL ratio in mice. Biochemical and Biophysical Research Communications, 511, 394–397. 30791983 10.1016/j.bbrc.2019.02.066

[phy270299-bib-0029] Kurgan, N. , Whitley, K. C. , Maddalena, L. A. , Moradi, F. , Stoikos, J. , Hamstra, S. I. , Rubie, E. A. , Kumar, M. , Roy, B. D. , Woodgett, J. R. , Stuart, J. A. , & Fajardo, V. A. (2019). A low‐therapeutic dose of lithium inhibits GSK3 and enhances myoblast fusion in C2C12 cells. Cells, 8, 1340. 31671858 10.3390/cells8111340 PMC6912290

[phy270299-bib-0030] L'Abbate, S. , Nicolini, G. , Marchetti, S. , Forte, G. , Lepore, E. , Unfer, V. , & Kusmic, C. (2023). Lithium treatment induces cardiac dysfunction in mice. International Journal of Molecular Sciences, 24(21), 15872. 10.3390/ijms242115872 37958854 PMC10650075

[phy270299-bib-0031] MacLennan, D. H. , & Kranias, E. G. (2003). Calcium: Phospholamban: A crucial regulator of cardiac contractility. Nature Reviews Molecular Cell Biology, 4, 566. 12838339 10.1038/nrm1151

[phy270299-bib-0032] Makarewich, C. A. , Munir, A. Z. , Schiattarella, G. G. , Bezprozvannaya, S. , Raguimova, O. N. , Cho, E. E. , Vidal, A. H. , Robia, S. L. , Bassel‐Duby, R. , & Olson, E. N. (2018). The DWORF micropeptide enhances contractility and prevents heart failure in a mouse model of dilated cardiomyopathy. eLife, 7, e38319. 10.7554/eLife.38319 30299255 PMC6202051

[phy270299-bib-0033] Michael, A. , Haq, S. , Chen, X. , Hsich, E. , Cui, L. , Walters, B. , Shao, Z. , Bhattacharya, K. , Kilter, H. , Huggins, G. , Andreucci, M. , Periasamy, M. , Solomon, R. N. , Liao, R. , Patten, R. , Molkentin, J. D. , & Force, T. (2004). Glycogen synthase kinase‐3beta regulates growth, calcium homeostasis, and diastolic function in the heart. The Journal of Biological Chemistry, 279, 21383–21393. 15020584 10.1074/jbc.M401413200

[phy270299-bib-0034] Morisco, C. , Zebrowski, D. , Condorelli, G. , Tsichlis, P. , Vatner, S. F. , & Sadoshima, J. (2000). The Akt‐glycogen synthase kinase 3beta pathway regulates transcription of atrial natriuretic factor induced by beta‐adrenergic receptor stimulation in cardiac myocytes. The Journal of Biological Chemistry, 275, 14466–14475. 10799529 10.1074/jbc.275.19.14466

[phy270299-bib-0035] Pansters, N. A. , Schols, A. M. , Verhees, K. J. , de Theije, C. C. , Snepvangers, F. J. , Kelders, M. C. , Ubags, N. D. , Haegens, A. , & Langen, R. C. (2015). Muscle‐specific GSK‐3beta ablation accelerates regeneration of disuse‐atrophied skeletal muscle. Biochimica et Biophysica Acta, 1852, 490–506. 25496993 10.1016/j.bbadis.2014.12.006

[phy270299-bib-0036] Richardson, T. , & Macaluso, M. (2017). Clinically relevant treatment considerations regarding lithium use in bipolar disorder. Expert Opinion on Drug Metabolism & Toxicology, 13, 1105–1113. 28965429 10.1080/17425255.2017.1386653

[phy270299-bib-0037] Sakata, S. , Lebeche, D. , Sakata, N. , Sakata, Y. , Chemaly, E. R. , Liang, L. F. , Tsuji, T. , Takewa, Y. , del Monte, F. , Peluso, R. , Zsebo, K. , Jeong, D. , Park, W. J. , Kawase, Y. , & Hajjar, R. J. (2007). Restoration of mechanical and energetic function in failing aortic‐banded rat hearts by gene transfer of calcium cycling proteins. Journal of Molecular and Cellular Cardiology, 42, 852–861. 17300800 10.1016/j.yjmcc.2007.01.003 PMC1945057

[phy270299-bib-0038] Vaduganathan, M. , Mensah, G. A. , Turco, J. V. , Fuster, V. , & Roth, G. A. (2022). The global burden of cardiovascular diseases and risk. Journal of the American College of Cardiology, 80, 2361–2371. 36368511 10.1016/j.jacc.2022.11.005

[phy270299-bib-0039] Vella, C. A. , & Robergs, R. A. (2005). A review of the stroke volume response to upright exercise in healthy subjects. British Journal of Sports Medicine, 39, 190–195. 15793084 10.1136/bjsm.2004.013037 PMC1725174

[phy270299-bib-0040] Weeks, K. L. , & McMullen, J. R. (2011). The athlete's heart vs. the failing heart: Can signaling explain the two distinct outcomes? Physiology (Bethesda), 26, 97–105. 21487028 10.1152/physiol.00043.2010

[phy270299-bib-0041] Whitley, K. C. , Hamstra, S. I. , Baranowski, R. W. , Watson, C. J. F. , MacPherson, R. E. K. , MacNeil, A. J. , Roy, B. D. , Vandenboom, R. , & Fajardo, V. A. (2020). GSK3 inhibition with low dose lithium supplementation augments murine muscle fatigue resistance and specific force production. Physiological Reports, 8, e14517. 32729236 10.14814/phy2.14517 PMC7390913

[phy270299-bib-0042] Yla‐Herttuala, S. (2015). Gene therapy for heart failure: Back to the bench. Molecular Therapy, 23, 1551–1552. 26442799 10.1038/mt.2015.158 PMC4817921

[phy270299-bib-0043] Young, D. B. (2010). Control of cardiac output. Morgan & Claypool Life Sciences. 21634064

